# Electrophysiological Studies into the Safety of the Anti-diarrheal Drug Clotrimazole during Oral Rehydration Therapy

**DOI:** 10.1371/journal.pntd.0004098

**Published:** 2015-09-25

**Authors:** Willem S. Lexmond, Paul A. Rufo, Edda Fiebiger, Wayne I. Lencer

**Affiliations:** Division of Gastroenterology and Nutrition, Boston Children’s Hospital, Harvard Medical School, Boston, Massachusetts, United States of America; University of California San Diego School of Medicine, UNITED STATES

## Abstract

**Background and Aims:**

Morbidity and mortality from acute diarrheal disease remains high, particularly in developing countries and in cases of natural or man-made disasters. Previous work has shown that the small molecule clotrimazole inhibits intestinal Cl^-^ secretion by blocking both cyclic nucleotide- and Ca^2+^-gated K^+^ channels, implicating its use in the treatment of diarrhea of diverse etiologies. Clotrimazole, however, might also inhibit transporters that mediate the inwardly directed electrochemical potential for Na^+^-dependent solute absorption, which would undermine its clinical application. Here we test this possibility by examining the effects of clotrimazole on Na^+^-coupled glucose uptake.

**Materials and Methods:**

Short-circuit currents (Isc) following administration of glucose and secretagogues were studied in clotrimazole-treated jejunal sections of mouse intestine mounted in Ussing chambers.

**Results:**

Treatment of small intestinal tissue with clotrimazole inhibited the Cl^-^ secretory currents that resulted from challenge with the cAMP-agonist vasoactive intestinal peptide (VIP) or Ca^2+^-agonist carbachol in a dose-dependent fashion. A dose of 30 μM was effective in significantly reducing the Isc response to VIP and carbachol by 50% and 72%, respectively. At this dose, uptake of glucose was only marginally affected (decreased by 14%, p = 0.37). There was no measurable effect on SGLT1-mediated sugar transport, as uptake of SGLT1-restricted 3-O-methyl glucose was equivalent between clotrimazole-treated and untreated tissue (98% vs. 100%, p = 0.90).

**Conclusion:**

Treatment of intestinal tissue with clotrimazole significantly reduced secretory responses caused by both cAMP- and Ca^2+^-dependent agonists as expected, but did not affect Na^+^-coupled glucose absorption. Clotrimazole could thus be used in conjunction with oral rehydration solution as a low-cost, auxiliary treatment of acute secretory diarrheas.

## Author Summary

In acute infectious diarrhea, the active secretion of Cl^-^ ions contributes to the secondary loss of Na^+^ and water from the intestine. Apical Cl^-^ secretion from intestinal epithelial cells is dependent upon cyclic nucleotide- and Ca^2+^-dependent intracellular signals and requires the concomitant transport of K^+^ through basolateral K^+^ channels for maintenance of an electroneutral state. Hence, when efflux of K^+^ in enterocytes is blocked, Cl^-^ secretion necessarily shuts down. The FDA-approved antifungal drug clotrimazole has been demonstrated to be a potent blocker of basolateral cAMP- and Ca^2+^-gated K^+^ channels in enterocytes, and therefore likely has therapeutic efficacy for secretory diarrheas. One important concern that could compromise its clinical applicability as a novel anti-diarrheal drug, however, is that clotrimazole might affect intestinal Na^+^-coupled glucose absorption, which constitutes the physiological basis of oral rehydration therapies and is thus critical for the efficacy of the current golden standard treatment for acute infectious diarrheal diseases. In this work, we demonstrate that clotrimazole effectively blocks Cl^-^ secretion in mouse intestine after stimulation with secretory stimuli, without affecting the capacity to take up Na^+^ and glucose. These results pave the way towards further clinical development of clotrimazole as a new pharmacologic strategy for acute diarrheal disease.

## Introduction

The discovery of oral rehydration solution (ORS) for the treatment of acute diarrheal disease is considered one of the greatest medical advances of the 20^th^ century [1]. Since its introduction in the late 1960’s, the worldwide promotion and distribution of ORS has led to a significant reduction in diarrheal disease-associated mortality [2]. Still, despite the high efficacy of ORS in replenishing isotonic fluid losses and preventing dehydration, diarrheal disease continues to be a leading cause of world-wide mortality, particularly in developing countries, in vulnerable populations such as children and the elderly, and especially in cases of natural (or man-made) disasters such as occurred following the recent earthquakes in Haiti and Nepal [2]. Furthermore, recurrent or prolonged infectious diarrhea in childhood is often associated with significant morbidity that impacts long-term growth and development [3,4]. As such, there remains a need for new and effective pharmacological therapies for the treatment of acute diarrheal disease [5,6].

In many cases of acute infectious diarrhea, the active secretion of salt and water contributes to the clinical disease. This depends primarily on the secretion of Cl^-^ anions into the intestinal lumen by enterocytes lining the intestinal crypt. The resulting increase in the lumenal concentration of Cl^-^ ions mediates a transepithelial electrochemical gradient, which drives the paracellular flux of Na^+^ and water into the intestinal lumen and thus accounts for the net loss of salt and fluid in diarrhea [7]. In many cases, inhibition of Na^+^ (re)absorption by blockade of the apical membrane Na^+^/H^+^-exchanger (NHE3) on absorptive enterocytes in intestinal villi contributes to a further loss of salts and water in the stool [8].

Intestinal Cl^-^ channels are operative under physiological conditions, but become abnormally activated in many diarrheal diseases by agonists that increase levels of intracellular cAMP, cGMP, or Ca^2+^. In the intestinal crypt, these second messengers open apical membrane Cl^-^ channels and basolateral membrane K^+^ channels, allowing efflux of Cl^-^ from enterocytes. These enterocytes are Cl^-^ ion-loaded through the action of the basolateral Na^+^-coupled Na^+^/K^+^/2Cl^-^ co-transporter NKCC1. Transport by NKCC1 of Cl^-^ into the cell is driven by the inwardly directed electrochemical Na^+^ gradient produced by the Na^+^/K^+^ATPase [7]. In all cases, the driving force for Cl^-^ uptake and secretion is maintained by the concomitant activation of basolateral membrane K^+^ channels that balance membrane potential and enable the Na^+^/K^+^/2Cl^-^ co-transporter to continue operating [7]. Without concomitant K^+^ efflux, the Cl^-^ secretory pathway shuts down.

The imidazole antifungal clotrimazole (CLT) blocks conductive K^+^ transport mediated by both cyclic nucleotide- and Ca^2+^-gated basolateral K^+^ channels [9]. This abolishes the driving force for Cl^-^ secretion in the intestine and, therefore, has therapeutic potential to reduce intestinal fluid loss. Indeed, proof-of-principle experiments in mouse and rabbit intestine, as well as human intestinal cell lines suggest the utility of CLT for treatment of secretory diarrheas [9,10].

Because of the high efficacy and widespread implementation of ORS in the treatment of diarrheal disease, it is paramount that novel anti-diarrheal agents show efficacy without disrupting rehydration therapy. Fundamental to the working of ORS is intestinal Na^+^-coupled glucose co-transport by members of the SGLT family [11]. These transporters, most prominently represented by SGLT1, are directly and functionally dependent on the electrochemical Na^+^-gradient maintained by the basolateral Na^+^/K^+^ATPase and basolateral K^+^ efflux to maintain cell potential during the absorptive process. There are many K^+^ channels on the basolateral membranes of enterocytes that allow for Na^+^/K^+^ATPase function and that are not responsive to cAMP and Ca^2+^. It has been shown *in vitro* that CLT can affect the Na^+^/K^+^ATPase [12] and in principle it might also inhibit other K^+^ channels affecting membrane potential in the absorptive state, thus inhibiting Na^+^-dependent solute absorption fundamental to ORS. To address this concern, we tested whether CLT inhibits Na^+^-dependent glucose uptake in the mouse intestine.

## Materials and Methods

### Ethics statement

All animals used in these experiments were kept and euthanized in accordance with Institutional Animal Care and Use Committee approved protocol number 13-06-2415R (IACUC, Boston Children’s Hospital), and in adherence to the National Research Council’s ‘Guide to the care and Use of Laboratory Animals’. Boston Children’s Hospital is accredited by AAALAC International and maintains appropriate Assurance of Compliance with the Office for Protection of Research Risk of the National Institutes of Health. Boston Children’s Hospital assurance number is A3303-01.

### Animals and reagents

Adult Balb/C mice (6–8 weeks of age) were bred in house and euthanized by CO_2_. Clotrimazole (C6019), glucose (G8270), mannitol (M4125), phloridzin dihydrate (P3449), 3-O-Methyl-D-glucopyranose (M4879), vasoactive intestinal peptide, and carbachol (C4382) were all obtained from Sigma-Aldrich (St. Louis, MO). Krebs-Henseleit buffer (KHB) was prepared at a pH of 7.6 with NaCl (118 mM), KCl (4.7 mM), CaCl_2_ (2.5 mM), MgSO_4_ (1.6 mM), NaHCO_3_ (24.9 mM), KH_2_PO_4_ (1.2 mM), and sodium pyruvate (2 mM).

### Tissue preparation

Following euthanasia with CO_2_, the small intestine of mice was resected and transferred to KHB on ice. At 6 cm from the pylorus, the jejunum was incised and 4 adjacent sections of approximately 2 cm were cut, avoiding Peyer’s patches, and gently flushed with KHB. Sections were opened longitudinally and mechanically stripped of *tunica serosa* and *tunica muscularis*. Opened sections were transferred onto Ussing chamber inserts with a 0.3 cm^2^ open surface area (Physiologic Instruments) and kept in KHB on ice while adjacent sections were prepared. Up to 3 sections of jejunum were used per mouse. Experiments consisted of the simultaneous analysis of up to 6 individual samples.

### Ussing chamber experiments

Inserts with jejunal tissues were mounted in Ussing chambers (Physiologic Instruments) and equilibrated in 10 ml pre-warmed, pre-oxygenated (95% O_2_ / 5% CO_2_) KHB, containing 10 mM glucose in the serosal chamber and 10 mM mannitol in the lumenal chamber. Electrophysiological measurements were performed with Ag-AgCl electrodes connected to salt bridges that were prepared from 3% agarose in 3 M KCl solution in plastic electrode tips (P2023-100, Physiologic Instruments). After 15 minutes of equilibration, transepithelial resistance was measured to assess tissue viability and complete stripping of muscle layer. Vital intestinal tissue was treated by the addition of CLT or ethanol vehicle to both serosal and lumenal chambers for 20 minutes, after which 10 mM glucose was added to the lumenal chamber. The secretagogues VIP and carbachol were added to the serosal chambers. Because current convention identifies the serosal (basolateral) bath as ground [13], both the absorption of positively charged Na^+^ ions, as well as the lumenal secretion of negatively charged Cl^-^ ions result in an increase in negative Isc. During the experiments, Isc measurements were collected every minute using Acquire software (Physiologic Instruments). ΔIsc was defined as the absolute change in Isc following challenge with the respective compound as follows: ΔIsc = Isc_t = x−_Isc_t = 0_, in which t = 0 represents the Isc immediately before each challenge and x varies with the investigated compounds as indicated per experiment. In some experiments, the maximal recorded change from baseline (Max ΔIsc) was shown for individual tissue samples in addition to the pooled Isc registration.

### Statistical analysis

Samples were compared with a two-sided student t test or ANOVA with Bonferroni’s correction for multiple testing where indicated at an alpha level of 0.05. Analyses were performed with Prism 5.0 (GraphPad Software, La Jolla, CA).

## Results

**Fig 1:**
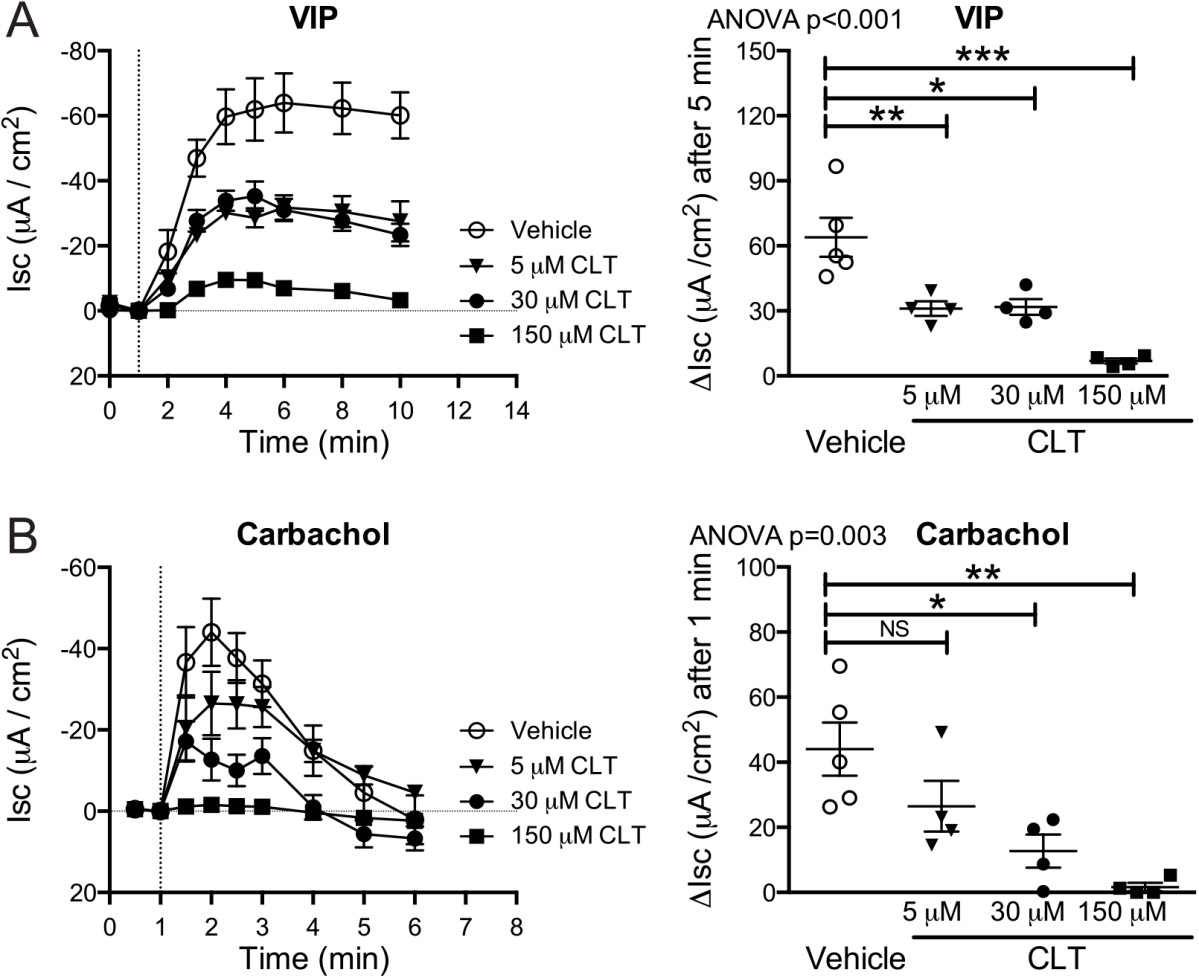
Clotrimazole inhibits the effect of secretagogues in a dose-dependent fashion. Jejunal tissue preparations were apically and basolaterally treated with 5, 30, and 150 μM clotrimazole (CLT) or vehicle control for 20 minutes in Ussing chambers and subsequently challenged with the cAMP-dependent secretagogue VIP (7.5 nm) or the Ca^2+^-dependent secretagogue carbachol (20 μM). Short-circuit current (Isc) registration and statistical analysis following VIP (**A**) and carbachol (**B**) administration to basolateral chamber at t = 5 and t = 1 respectively show dose-dependent inhibition of secretory Isc by CLT. Pooled data from 3 independent experiments, with a total of 4 intestinal sections per condition. Error bars represent the SEM. *p<0.05; ** p<0.01; ***p<0.001; NS: not significant as determined by ANOVA with Bonferroni’s multiple comparison test.

To confirm the previously described inhibitory effect of CLT on intestinal Cl^-^ secretion induced by cAMP- or Ca^2+^-dependent agonists [9,10], we compared the short-circuit current (Isc) observed before and after challenge with vasoactive intestinal peptide (VIP) or carbachol of mouse jejunal tissues treated with 0, 5, 30, or 150 μM CLT in Ussing chambers. CLT treatment resulted in a dose-dependent decrease in Isc induced by both secretagogues, consistent with reduced secretion of Cl^-^ anions by enterocytes (**Fig 1A and 1B**). With this limited sample number, we did not observe a difference in VIP-induced responses between treatment with 5 or 30 μM CLT, but a dose of 30 μM was found to be effective in significantly reducing the Isc response to both VIP and carbachol by 50% and 72%, respectively. Based on these studies, together with our well delineated dose dependency studies of CLT in human intestinal cell lines [9], this dose was used in all subsequent studies.

**Fig 2:**
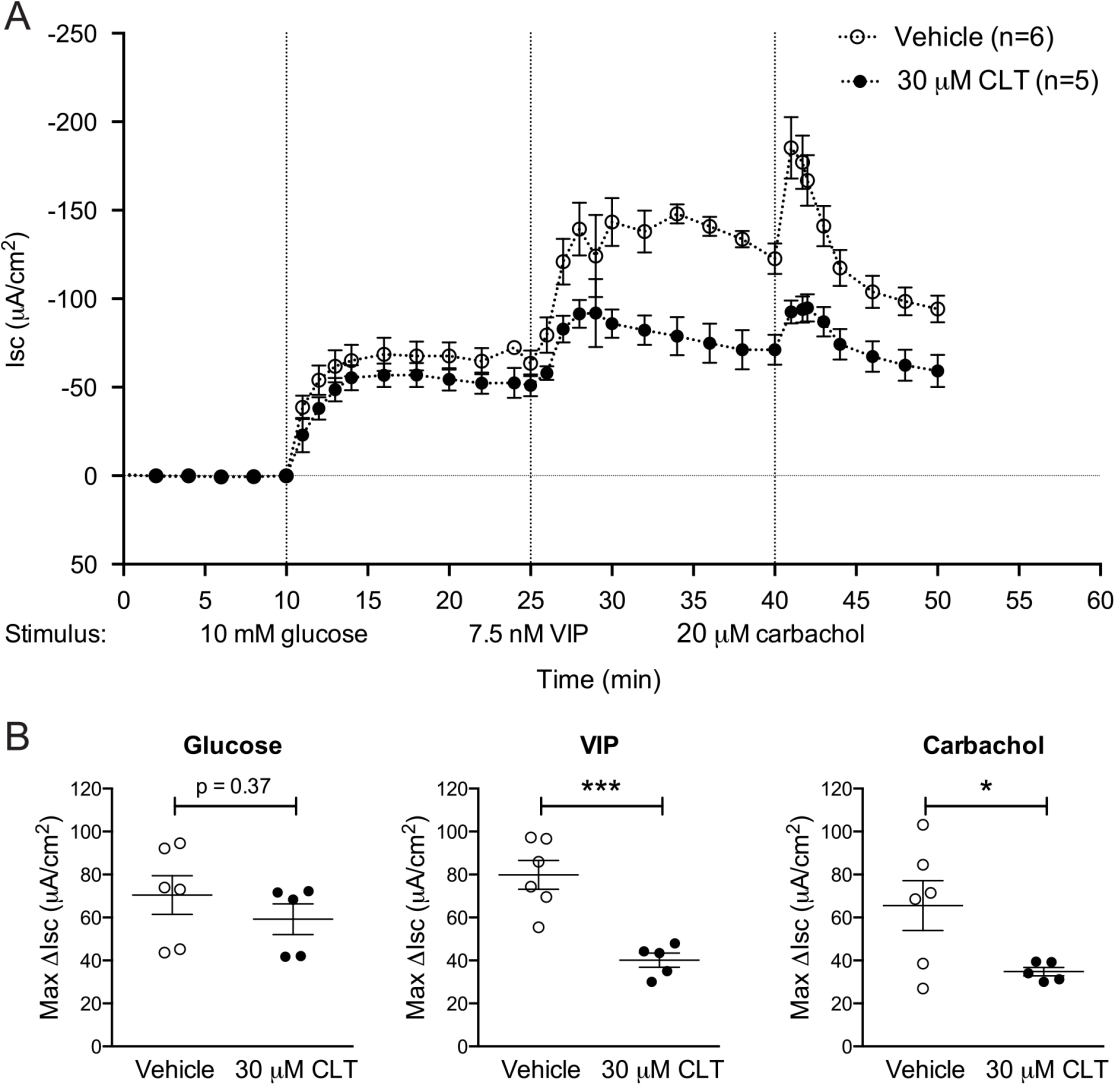
CLT blocks the action of secretagogues without impairing glucose uptake. **(A)** Isc registration of vehicle (n = 6) or CLT-treated (n = 5) tissues that were sequentially challenged with 10mM glucose apically (t = 10), and 7.5 nM VIP (t = 25) and 20 μM carbachol (t = 40) basolaterally. (**B-D**) Summary statistics of the maximal recorded change in Isc (ΔIsc) within 10 minutes of challenge with the investigated compound from baseline Isc before challenge. Pooled data from 2 independent experiments. Error bars represent the SEM. *p<0.05; ***p<0.001 as assessed by student t-test.

To test if CLT interferes with Na^+^-coupled glucose uptake in the small intestine, we determined the effect of CLT on changes in Isc caused by the addition of glucose to the lumenal (apical) chamber of mouse jejunum mounted in Ussing chambers. In both CLT and vehicle-treated tissue, the addition of lumenal glucose resulted in a rapid increase in negative Isc, signifying an increase in the uptake of Na^+^ cations (**Fig 2A and 2B**, and individual tracings are shown in supplemental S1 **Fig**). Fifteen minutes after the addition of glucose to the lumenal reservoir, the intestinal tissues were challenged basolaterally with the cAMP-dependent agonist VIP and Ca^2+^-dependent agonist carbachol to confirm that CLT inhibited cAMP and Ca^2+^-induced Cl^-^ secretion under these conditions (**Fig 2C and 2D**).

**Fig 3:**
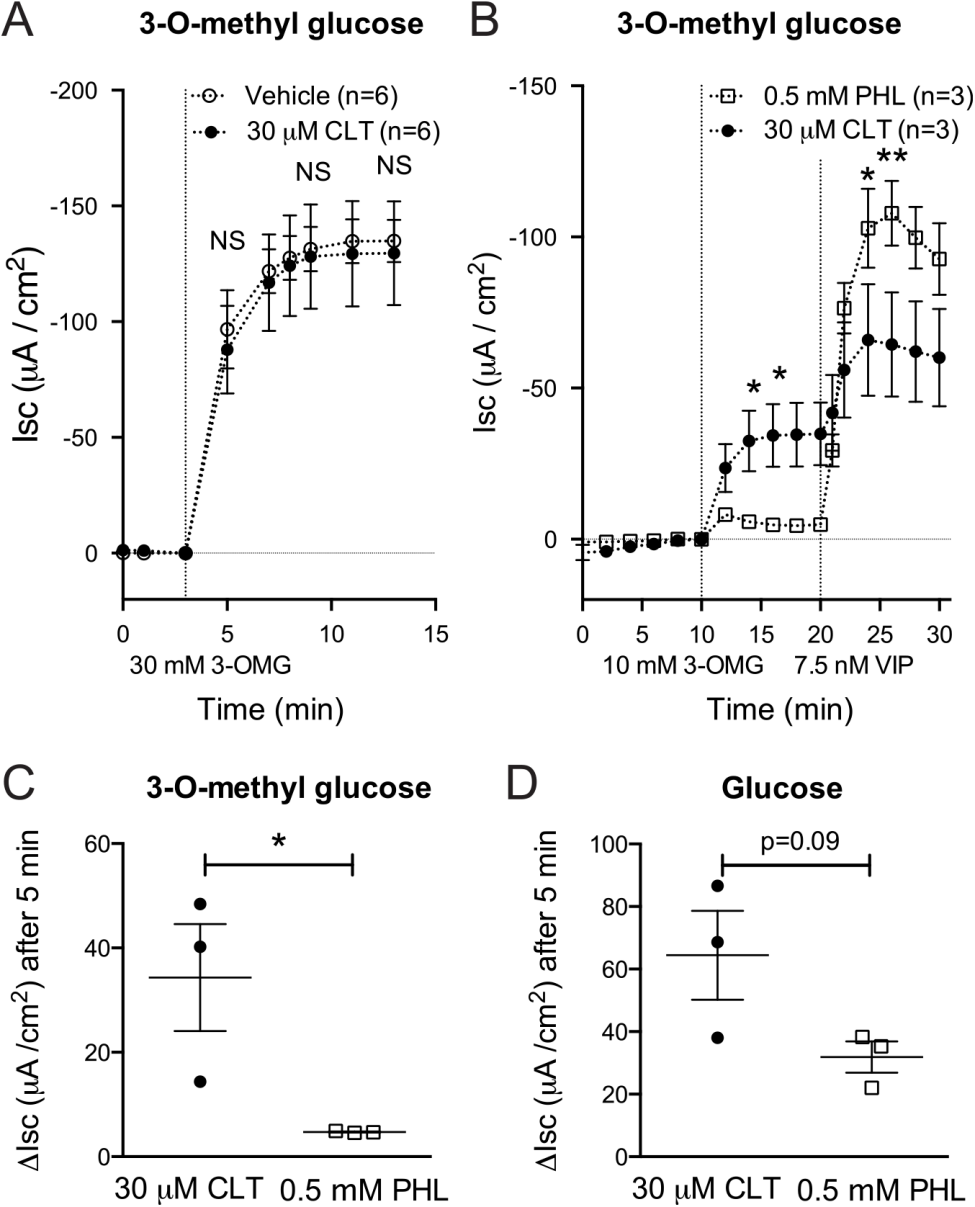
Clotrimazole treatment does not inhibit the function of SGLT1. **(A)** Equivalent changes in Isc following apical administration of 30 mM 3-O-methyl glucose (3-OMG) rule out an inhibitory effect on SGLT1 from 30 μM CLT. (**B**) Phloridzin (PHL)-mediated SGLT1 blockade decreases Isc changes in response to 3-O-methyl glucose but does not affect Cl^-^ secretion in response to VIP, with individual data points at t = 5 depicted in (**C**). (**D**) ΔIsc elicited by apical administration of 10 mM glucose is partially blocked by PHL treatment. Pooled data from 2 independent experiments in A and single experiments in B-D. Error bars represent SEM. *p<0.05; **p<0.01; NS not significant as assessed by student t-test on the ΔIsc following addition of described stimulus at the indicated time points.

Though well within normal variation, we did note a small reduction in glucose-induced ΔIsc in CLT-treated tissues when compared to untreated jejunum (decreased 14%, p = 0.37, **Fig 2B**). Thus, to specifically test the effect of CLT on the function of SGLT1, the dominant Na^+^-coupled glucose transported in the intestine [11], we repeated the experiment depicted in Fig 2 with the SGLT1-specific ligand 3-O-methyl glucose. High-dose (30 mM 3-O-methyl glucose) lumenal challenge resulted in a rapid and predictably more pronounced solute-induced ΔIsc than observed with 10 mM glucose. Even with this greater signal size, no difference was observed between CLT and vehicle-treated tissue (**Fig 3A**, 98% vs. 100%, p = 0.90); while secretory responses to VIP and carbachol again remained significantly inhibited under these conditions (S2 Fig). These studies indicate that CLT has no detectable effect on Na-coupled glucose absorption through SGLT-1.

To further confirm that the changes in Isc after glucose or 3-O-methyl glucose administration were mediated by SGLT-1 (or related family members), we performed separate experiments in which tissues were treated with phloridzin, which blocks SGLT1 and to lesser extent other SGLT1 isoforms [14]. In the case of intestinal tissues exposed to 3-O-methyl glucose, the solute-induced ΔIsc was almost completely inhibited by pre-treatment with phloridzin (**Fig 3B and 3C**). Phloridzin-treated intestinal tissues continued to respond normally with VIP (cyclic nucleotide)-mediated Cl^-^ secretion, showing that phloridzin treatment did not affect the viability of intestinal tissue sections and that SGLT1 therefore was the source of the 3-O-methyl glucose-induced ΔIsc (**Fig 3B and 3C**). In the case of intestinal tissues exposed to glucose (**Fig 3D**), the solute-induced ΔIsc was also inhibited, and to approximately the same extent as in tissues exposed to 3-O-methyl glucose (about 30 μAmp), but the total ΔIsc induced by glucose was greater and inhibition by phlorizin was incomplete. Thus, SGLT1 explains a large fraction of Na^+^-coupled solute absorption (ΔIsc) induced by glucose; but the results also implicate transport through other SGLT family members known to be expressed in the intestine [11] that are less responsive to phloridzin, such as SGLT4 [14,15]. Combined, these studies confirm that Isc changes in response to glucose stimulation reflect Na^+^-coupled glucose transport, and demonstrate that SGLT1 is unaffected by CLT treatment, further strengthening the rationale for clinical applications of CLT in treatment of secretory diarrhea.

## Discussion

In this report we have assessed whether the K^+^-channel blocker CLT affected Na^+^-coupled glucose uptake in the intestine, which could be raised as an important concern for use of this drug in the treatment of secretory diarrheas. Our results demonstrate that Na^+^-coupled glucose uptake is not affected by CLT when administered in doses that effectively reduced both cAMP- and Ca^2+^-dependent chloride secretion. This strengthens the indications for use of CLT as an adjunct treatment in the management of diarrheal diseases of diverse etiologies.

The experiments presented here directly addressed the concern that the blocking effect of CLT on cAMP- and Ca^2+^-activated basolateral K^+^ channels in the enterocyte might disrupt Na^+^-coupled glucose uptake either by interfering with the maintenance of the intracellular Na^+^ electrochemical gradient established by the Na^+^/K^+^ ATPase, or by direct inhibition of SGLT1. Inhibition of Na^+^/K^+^ ATPase has previously been found *in vitro* using membrane fragments at a half-inhibiting concentration of 24–30 μM [12]. Similarly, direct inhibition by CLT of gastric H^+^/K^+^ ATPase has been documented [16]. Inhibition of these ATPases, however, is partial, even at high doses of CLT (100 μM) [12,16]. Furthermore, CLT inhibition of H^+^/K^+^ ATPase was pH-dependent [16]. In contrast to these studies, we observed that treatment of small intestinal tissue *ex vivo* with 30 μM CLT did not reduce Na^+^-coupled glucose uptake, suggesting that any effects of CLT on Na^+^/K^+^ ATPase in intact intestine *ex vivo* were not sufficient to interfere with effective glucose uptake. As such, our results are in line with previous *in vivo* studies in both mice and humans using prolonged oral treatment with CLT that showed normal growth and no evidence of toxicity [10,17], suggesting the effects of CLT on Na^+^/K^+^ ATPase are unlikely to be physiologically relevant. Our experiments have furthermore ruled out that CLT directly inhibits SGLT1. Even at doses of 150 μM, which is unlikely to be attained in patients due to the relative insolubility of CLT, there was only a modest apparent decrease (39%, n = 4) of Na^+^-glucose absorption while cAMP- and Ca^2+^-dependent Cl^-^ secretion was nearly completely abrogated.

With respect to potential clinical applications, we note that current FDA approved formulations of CLT are restricted to 1% topical/vaginal creams or solutions, and 10 mg lozenges for the treatment of oropharyngeal candidiasis. Previous studies, however, demonstrated that oral CLT is adequately absorbed, reaches peak serum values after 3 hours, and is metabolized primarily in the liver and excreted mostly in the feces [18]. In a trial with adult patients suffering from sickle cell anemia, a daily dose of 20 mg/kg in two tablets resulted in plasma levels <1 μM and was well-tolerated [19]. A small pediatric study reported no adverse events after CLT treatment with 100 mg/kg/day in 4 oral doses for 14–63 days [20]. We propose the use of CLT in treatment of acute diarrheal disease as an additive to currently available ORS solutions, though other forms of oral administration (e.g. tablets or capsules) are feasible as well. Our target concentration of 30 μM can be achieved by dissolving 10.3 mg CLT per liter of ORS. When administered in this way, the daily dose is unlikely to exceed 10 mg/kg bodyweight and thus remains far below daily doses that have previously been established to be safe [19,20]. Furthermore, direct addition of CLT to ORS establishes a very straightforward dosing schedule, since patients with the most severe diarrhea will require more ORS and will thus receive a higher daily dose of CLT. Conversely, CLT treatment will be tapered in parallel with reduced ORS requirement during the recovery phase. In the design of such formulations, it is important to recognize that CLT has poor solubility in water, measured by some to be as low 5.6 μg/ml (16 μM) [21]. Although our data demonstrated therapeutic efficacy with concentrations three times below this threshold, solubility and bioavailability of CLT could be enhanced by inclusion with β-cyclodextrin [22,23] or solid dispersions [21]. Lastly, it should be recognized that CLT has the potential to interact with other drugs that are metabolized through CYP3A [24].

In summary, our studies provide evidence that CLT can reduce cAMP- and Ca^2+^-dependent Cl^-^ secretion without affecting the ability of the enterocyte to absorb glucose, salts, and water. CLT is already an FDA-approved drug in widespread use as an anti-fungal, with considerable pharmacokinetic and toxicological data available. It is an ideal candidate for development as a novel, inexpensive anti-diarrheal agent. We suggest that combination treatment of CLT along with ORS could provide further gains in reducing mortality and morbidity caused by secretory diarrheas.

## Supporting Information

S1 Fig Isc tracings of individual samples treated with either CLT or vehicle control. Isc recordings are demonstrated for individual intestinal sections treated with 30 μM CLT (red tracings, n = 5) or vehicle control (blue tracings, n = 6) in response to the stimuli denoted on the x-axis.
(EPS)

S2 Fig Significant inhibition of secretagogue-induced current in CLT treated tissue. Isc tracings following VIP (7.5nM) or carbachol (20 μM) stimulation of intestinal tissue treated with CLT (n = 6) or vehicle control (n = 6) that had been previously exposed to 3-O-methyl glucose. Error bars represent SEM. *p<0.05; **p<0.01; as assessed by student t-test.
(EPS)
